# A hormone, a channel and a brake: melatonin orchestrates calcium signaling for aphid resistance

**DOI:** 10.1093/plphys/kiag506

**Published:** 2026-07-15

**Authors:** Marcella Teixeira

**Affiliations:** Assistant Features Editor, Plant Physiology, American Society of Plant Biologists, Rockville, MD 20855, United States; Hermiston Agricultural Research and Extension Center, Oregon State University, Hermiston, OR 97838, United States

Aphids are among the most destructive pests worldwide. Beyond the direct damage caused by their piercing-sucking mouthparts (Burd and Burton 1992), these small insects can vector a diverse range of plant viruses (Züst and Agrawal 2016) and promote sooty mold colonization through their sugary honeydew secretions (Wilkinson et al. 1997). Understanding how plants naturally resist aphid attack is therefore of considerable agronomic importance. Plants perceive aphids primarily through salivary proteins, which trigger cell-surface receptor signaling. This includes calcium (Ca^2+^) influx, reactive oxygen species accumulation, activation of defense genes, and secondary metabolite biosynthetic enzymes (Züst and Agrawal 2016).

One of these metabolites is melatonin (N-acetyl-5-methoxy-tryptamine), a regulatory molecule that impacts aphid mortality and development (Gao and Hardie 1997). Exogenous application of melatonin was recently shown to enhance resistance against aphids in wheat, tobacco, and watermelon (Zhou et al. 2021; Song et al. 2022; Su et al. 2023). In watermelon, this enhanced resistance is accompanied by increased activities of the defense-related enzymes chymotrypsin inhibitor, trypsin inhibitor, polyphenol oxidase (PPO), and phenylalanine ammonia-lyase, and lignin accumulation (Su et al. 2023).

Melatonin is involved in biotic and abiotic responses through modulation of Ca^2+^ signal transduction pathways (Chang et al. 2021, 2025). Notably, cyclic nucleotide-gated ion channels (CNGCs) are Ca^2+^ permeable channels that can interact with calmodulin 7 (CaM7) to mediate melatonin-induced Ca^2+^ influx (Chang et al. 2025). While CNGC20 mediates melatonin-induced cytosolic Ca^2+^ accumulation and influx, CaM7 inhibits exogenous melatonin-induced cytosolic Ca^2+^ (Chang et al. 2025). Despite the available information, little is known about the molecular pathway linking endogenous melatonin and Ca^2+^ signaling on plant resistance to aphids.

In this issue of *Plant Physiology*, Li et al. (2026) fill this gap by dissecting the signaling cascade that connects melatonin to aphid resistance in watermelon, placing cytosolic Ca^2+^ at the center. The study begins by establishing that aphid infestation itself triggers melatonin biosynthesis. Within 3 h of aphid attack, expression of *caffeic acid O-methyltransferase 1* (*ClCOMT1*) increases in watermelon, accompanied by a rise in endogenous melatonin levels.

This upregulation was followed by increased activities of trypsin inhibitor and PPO and, later, by an increase in chymotrypsin inhibitor, PPO, and lignin at 12 h postinfestation, consistent with melatonin acting upstream of these responses. These results showed that aphid feeding results in increased melatonin biosynthesis and activities of defense-related enzymes. To address the effect of endogenous melatonin levels and aphid resistance, the authors compared transgenic watermelon overexpressing *ClCOMT1* with the CRISPR/Cas9-generated knockout mutant *clcomt1*. *ClCOMT1*-OE showed enhanced resistance to aphids, with reduced aphid population and fecundity, increased defense enzyme activities, and higher lignin deposition. *clcomt1* showed the opposite phenotype, providing genetic evidence consistent with endogenous melatonin positively regulating aphid resistance.

Next, to investigate the role of Ca^2+^ signaling in melatonin-induced resistance, Li et al. (2026) used fluorescent probes in watermelon protoplasts and showed that aphid salivary proteins trigger a rapid rise in cytosolic calcium, a response amplified in *ClCOMT1*-OE protoplasts and attenuated in *clcomt1* protoplasts. To further explore the role of Ca^2+^ in ClCOMT1-induced resistance, the authors used different compounds to interfere with calcium signaling: CaCl_2_ (increases the extracellular concentration of Ca^2+^), A23187 (a chemical compound that facilitates Ca^2+^ influx across the plasma membrane), LaCl_3_ (a Ca^2+^ influx channel inhibitor), and EGTA (a Ca^2+^ chelator). CaCl_2_ treatment resulted in increased resistance to aphids, accompanied by higher lignin levels and defense-related enzyme activities. CaCl_2_ and A23187 treatment restored aphid resistance in the *clcomt1* mutant. Consistently, LaCl_3_ and EGTA pretreatments reduced the aphid resistance phenotype conferred by *ClCOMT1*-OE. These results support a role for Ca^2+^ in melatonin-induced defense responses against aphids.

The final piece of this puzzle is the molecular machinery connecting ClCOMT1 to Ca^2+^ influx. In watermelon, ClCNGC20 interacts with ClCaM7 to mediate melatonin-induced calcium signaling during cold stress, reducing calcium channel activity (Chang et al. 2025). Li et al. (2026) show that *ClCNGC20* is upregulated in *ClCOMT1*-OE and downregulated in *clcomt1* plants after aphid infestation, suggesting its regulation by melatonin in response to aphid infestation. Silencing *ClCNGC20* in *ClCOMT1*-OE significantly reduced accumulation of cytosolic Ca^2+^ in protoplasts treated with aphid salivary proteins and compromised the plants' enhanced aphid resistance. Conversely, *ClCaM7* was downregulated by melatonin in *ClCOMT1-*OE and upregulated in *clcomt1* plants, the opposite of *ClCNGC20*. Silencing *ClCaM7* in the *clcomt1* mutant restored cytosolic Ca^2+^ accumulation and aphid resistance.

The opposing roles of ClCNGC20 and ClCaM7 were further validated by their heterologous overexpression in *Arabidopsis*. *Arabidopsis* overexpressing *ClCNGC20* displayed elevated cytosolic Ca^2+^ and enhanced aphid resistance, whereas plants overexpressing *ClCaM7* showed reduced cytosolic Ca^2+^ and susceptibility.

Taken together, these findings reveal a 2-pronged mechanism (Fig. 1): upon aphid attack, watermelon plants upregulate the expression of *ClCOMT1* and increase melatonin content. Melatonin induces *ClCNGC20* expression, an increase in cytosolic Ca^2+^, and further activation of calcium signaling and increased resistance (Fig. 1a). The increase in melatonin content also inhibits *ClCaM7* expression, releasing the brake on ClCNGC20 channel activity (Fig. 1b). The resulting Ca^2+^ signal activates downstream defensive responses, including lignin deposition and defense-related enzyme activities (Fig. 1).

**Figure 1:**
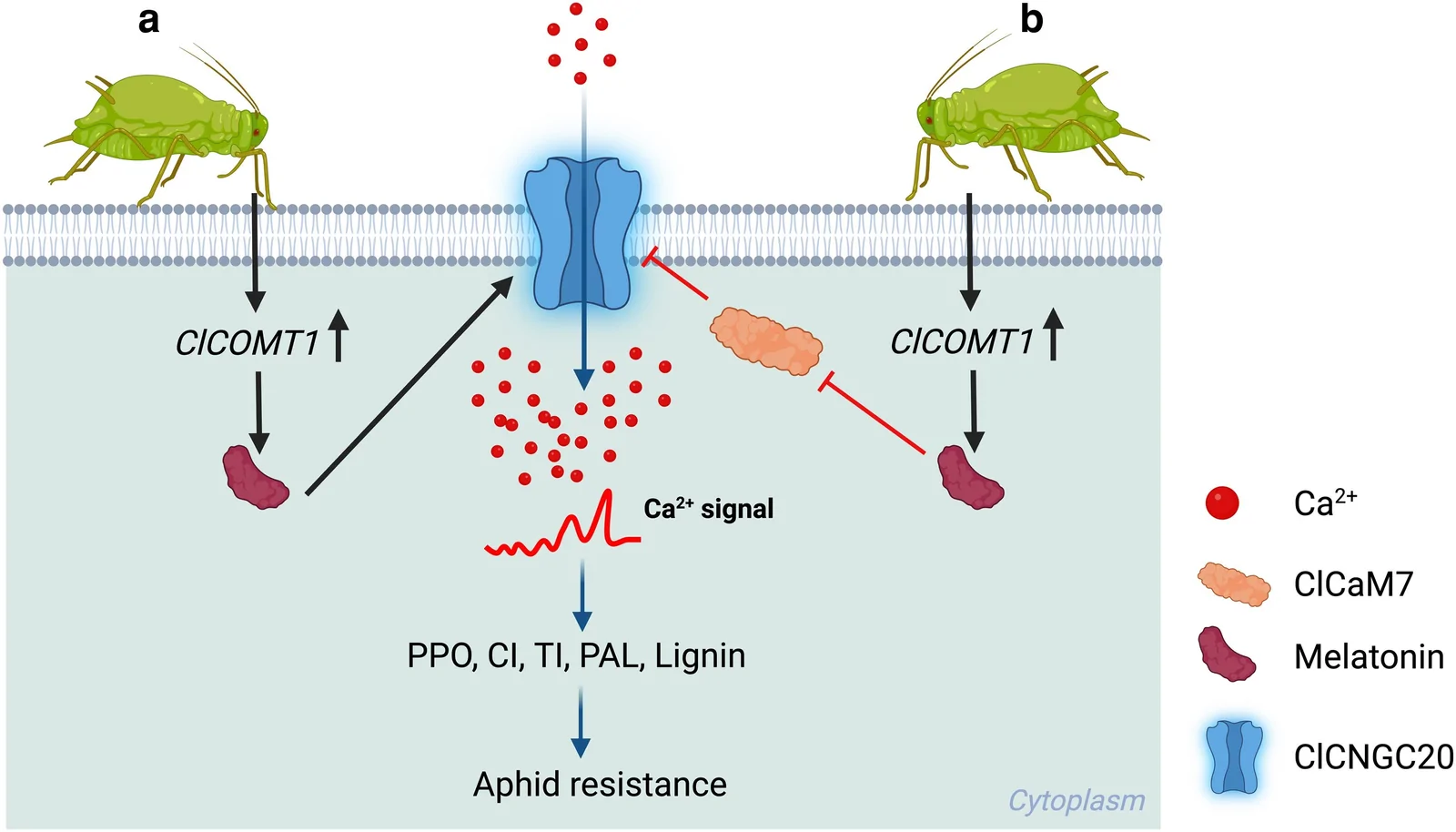
Working model for ClCNGC20 and ClCaM7 mediating melatonin-induced Ca^2+^ signaling and aphid resistance in plants. Upon aphid attack, watermelon upregulates ClCOMT1, which results in increased endogenous melatonin. (a) Melatonin increase induces upregulation of ClCNGC20, and a transient increase in cytosolic calcium. (b) Melatonin increase inhibits the expression of ClCaM7, alleviating its effect on inhibition of the ClCNGC20 channel. Figure adapted from Li et al. (2026).

The work of Li et al. (2026) advances our understanding of plant-aphid interactions by placing the melatonin signaling network upstream of Ca^2+^-dependent immunity through a defined molecular module. The identification of *ClCaM7* as a negative regulator is particularly noteworthy: silencing *ClCaM7* in the susceptible *clcomt1* mutant is sufficient to rescue aphid resistance, offering a promising strategy for engineering resistance in susceptible cucurbit varieties without requiring transgenic melatonin overproduction. Given that Ca^2+^ signaling is broadly implicated in plant immunity, it will be interesting to determine whether this ClCNGC20-ClCaM7 module also contributes to resistance against other pathogens or pest types. Additionally, the protoplasts system employed in the investigation also provides a platform for identifying the specific salivary elicitors recognized by plant receptors and the plant receptors themselves, questions that remain largely unanswered in plant-aphid biology.

**Recent related articles published in *Plant Physiology***

Dong X, Sui C, Wang K, Chen W, Li Y, Kear P J, Chang S, Lv D, Jian H. Melatonin enhances salt tolerance by inducing *StMYB55* to improve flavonoid accumulation in potato. *Plant Physiology* 2026:201(1), kiag168, https://doi.org/10.1093/plphys/kiag168. **The authors show that a melatonin-mediated transcriptional complex activates flavonoid accumulation in potato, protecting potato from salt stress.**

Muhammad I, Yang L, Ahmad S, Farooq S, Khan A, Muhammad N, Ullah S, Adnan M, Ali S, Liang Q P, Zhou X B. Melatonin-priming enhances maize seedling drought tolerance by regulating the antioxidant defense system. *Plant Physiology* 2023:191(4), 2301–2315, https://doi.org/10.1093/plphys/kiad027. **The authors show melatonin priming improves drought tolerance in maize seedlings alleviating the negative effects or reactive oxygen species.**

Nehela Y, Killiny N. Melatonin is involved in citrus response to the pathogen huanglongbing via modulation of phytohormonal biosynthesis. *Plant Physiology* 2020: 184(4), 2216–2239, https://doi.org/10.1104/pp.20.00393. **The authors show that Huanglongbing infection and *Diaphorina citri* infestation enhance melatonin biosynthesis and salicylic acid levels in citrus plants, revealing a role for melatonin-mediated defense against Huanglongbing.**

Shan Q, Zhao D, Cao B, Zhu X, Wang C, Deng L, Li C, Zhang Y, Shi Q, Gong B. Jasmonic acid and nitric oxide orchestrate a hierarchical melatonin cascade for *Botrytis cinerea* resistance in tomato. *Plant Physiology* 2025:197(3), kiaf078, https://doi.org/10.1093/plphys/kiaf078. **The authors show jasmonic acid and nitric oxide enhance COMT-mediated melatonin biosynthesis during *Botrytis cinerea* infection in tomato.**

Wang Z, Li S, Zhao T, Yu H, Wang Q, Wan Z, Wei C, Ma J, Zhang Y, Zhang X, Li H. NO-dependent H_2_O_2_ signaling regulates melatonin-induced resistance to *Fusarium oxysporum* f. sp. *niveum* race 1 in watermelon. *Plant Physiology* 2025:199(3), kiaf564, https://doi.org/10.1093/plphys/kiaf564. **The authors show the relevance of nitric oxide-dependent H_2_O_2_ signaling for melatonin-induced defense responses in watermelon against *Fusarium oxysporum* f. sp. *niveum*.**

## Data Availability

No data were generated in this study.
